# Effect of Rehabilitation on Recurrent Shoulder Dislocation Post-Latarjet Procedure: A Case Report

**DOI:** 10.7759/cureus.32804

**Published:** 2022-12-21

**Authors:** Gayatri S Kaple, Shivani R Uttamchandani, Pratik Phansopkar

**Affiliations:** 1 Musculoskeletal Physiotherapy, Ravi Nair Physiotherapy College, Datta Meghe Institute of Medical Sciences, Wardha, IND; 2 Musculoskeletal Physiotherapy, Ravi Nair Physiotherapy College, Datta Meghe Institute of Medical Sciences., Wardha, IND

**Keywords:** recurrent shoulder dislocation, geometry, glenohumeral articulation, latarjet procedure, pain

## Abstract

Recurrent shoulder dislocation is often induced as a result of a traumatic situation that results in structural disease; however, a small subset of individuals suffer symptomatic recurrent shoulder instability without trauma. The glenohumeral articulation geometry offers a lot of flexibility at the sacrifice of inherent stability. The commonly dislocated joint is the shoulder joint as it has intrinsic instability that can lead to repeated dislocations. The procedure is designed to minimize the probability of problems. Physiotherapy is essential for regaining patients' mobility and enhancing their quality of life. A 36-year-old patient visited Acharya Vinoba Bhave Rural Hospital with complaints of pain graded 7/10 on the numerical pain rating scale and injury to the right shoulder joint after which there was repeated shoulder dislocation after a fall because of epilepsy five years back. The patient was diagnosed with recurrent shoulder dislocation which occurred approximately 50 times in a year as of January 24, 2022. The patient was managed with a Latarjet procedure on January 28, 2022. A well-planned physical therapy intervention significantly improved their overall status of health along with their ability to perform daily activities efficiently in post-operative patients with recurrent shoulder dislocation.

## Introduction

The shoulder joint is formed by the humerus, clavicle, and scapula. The humerus head should be present in the center of the glenoid fossa. It thus permits entire joint surfaces to be positioned consistently. Furthermore, the response force of the glenohumeral joint is confined inside the glenoid arc. However, a gross glenohumeral joint resultant force has been compromised as in the situation of a shoulder dislocation. As a consequence, its humeral head is displaced out of the articular surface [1]. Instability is a frequent condition that affects the shoulder, in the growing population. Most cases of shoulder dislocations are caused by trauma, and if the injury causes structural damage, it can lead to recurrent instability. Surgery is frequently recommended to restore the damaged structures in this group of individuals, especially if they are young and active. In the absence of trauma, a tiny percentage of individuals experience recurrent shoulder instability [2]. This is a more challenging situation to handle, and surgery is typically not indicated. The focus of therapy is conservative treatment in physiotherapy based on exercise to restore muscular strength, proprioception, and muscle coordination within the shoulder girdle [3].

The shoulder joint is most prone to dislocation within the body. Anterior shoulder dislocations are most frequently clinically diagnosed. In this condition, the arms of patients often appear internally rotated and adducted, revealing a lack of appropriate deltoid contour [4]. Here, we present a 36-year-old male with recurrent shoulder dislocation, which has been treated with a Latarjet procedure; the risk factors for complications and treatment failure after Latarjet surgery include the atraumatic mechanism of primary dislocation, anterior glenohumeral joint destabilization that can be treated surgically through repeated instabilities, dislocation arthropathy, and postoperative restriction of the external rotation. This coracoid graft is attached toward the anteroinferior aspect of the glenoid between three and five o'clock. However, the Latarjet surgical procedure reattached the labrum among the ordinary glenoid and the bone transplant. Based on one analysis, 2% had anterior glenohumeral instability. The glenohumeral joint is the most often misplaced synovial joint in the human body. Forceful adduction and external rotation of the shoulder joint can cause instability [5].

## Case presentation

Here, the case of a 36-year-old male laborer who had been visiting the tertiary care rural hospital for the past five years with a primary complaint of shoulder pain and frequent shoulder dislocation is reported. The patient was apparently alright five years back when he allegedly fell on the ground sustaining an injury to the right shoulder. He later noticed that his shoulder was displaced from its original position. Then, he visited the nearby hospital in his village where an X-ray and magnetic resonance imaging (MRI) of the shoulder joint were done. In the hospital, he was managed by externally fixating (reducing) by pushing the shoulder inside and was suggest to immobilize the shoulder joint for 15 days. Then, a few months after the incident, the patient experienced the same problem repeatedly, and he reduced it by pushing the shoulder inside. However, he started experiencing similar episodes of shoulder dislocation especially after raising his hand overhead and hence had difficulty in daily activities such as combing hair and sleeping with his hand overhead. The patient indicated shoulder dislocation around 50 times in five years. The patient also had a history of seizures for five years accompanied by shoulder dislocation after which he used to experience unbearable pain in the shoulder joint. The patient was then referred to Acharya Vinoba Bhave Rural Hospital (AVBRH) for the management of the same and was admitted to the hospital, and all investigations were done. The patient was managed surgically by soft tissue reconstruction of the right shoulder with a Latarjet procedure. On postoperative day 1, he was referred to the physiotherapy department for additional care.

Clinical findings

**Figure 1:**
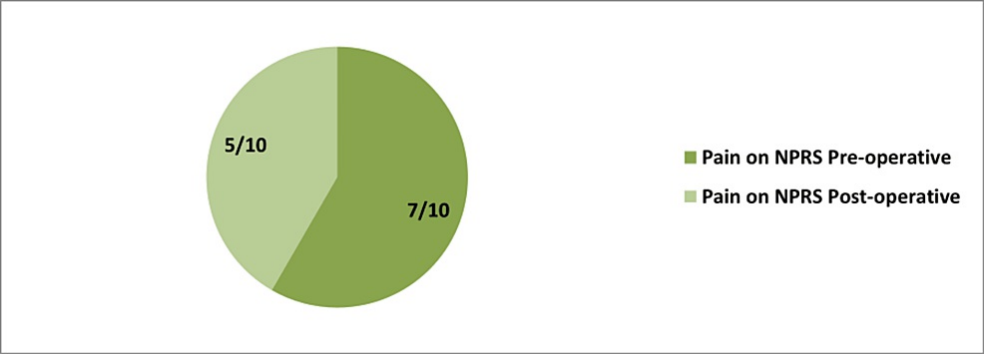
Pie shows pre-op on the left and post-op on the right of patient's shoulder pain on the NPRS. NPRS: Numerical pain rating scale

On general examination, the pulse rate was 76 beats/min, blood pressure was 126/82 mmHg, and height and weight were 179 cm and 60 kg, respectively, according to which body mass index calculated was 18.7. The patient experienced pain of 5/10 and 7/10 pre-operative and post-operative, respectively, on the numerical pain rating scale. On local examination, the patient was examined in a supine position, and on inspection, the right shoulder joint overlying skin appeared normal, and no scars and sinuses were present. On palpation, the patient's range of motion was terminally painful, active finger movement was present, and radial pulsation was present. Tenderness of grade 2 was palpated, and there was a spasm in the rotator cuff muscle (Figure 1).

Timeline

All the events that occurred during patient hospitalization are shown in Table 1.

**Table 1:** Events that occurred while the patient was hospitalized. AVBRH: Acharya Vinoba Bhave Rural Hospital

Date of admission to AVBRH	December 23, 2021
Date of diagnosis of shoulder dislocation	December 24, 2021
Date of operation	January 28, 20222
Date of physiotherapy referral	January 29, 2022
Date of discharge	February 9, 2022

Diagnostic assessment

Many tests were conducted on the patient, including complete blood count, a liver function test, a kidney function test, a rapid blood sugar test, and blood group determination. However, no aberrant discoveries were made.

Shoulder X-ray 

**Figure 2:**
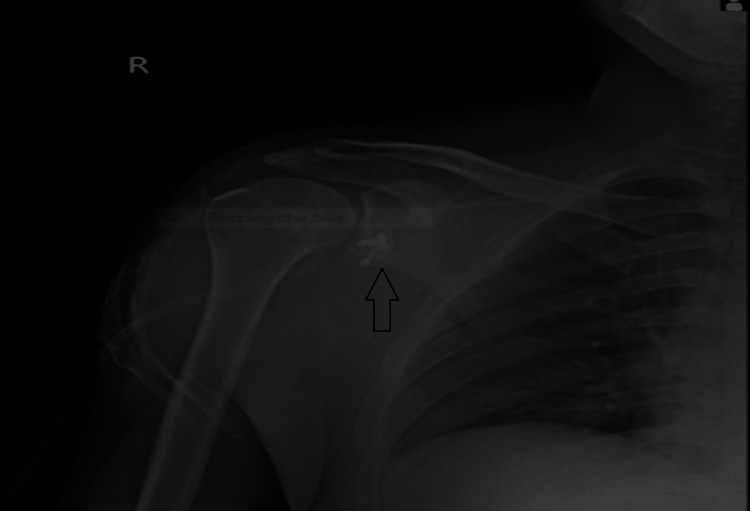
Post-operative X-ray in AP view showing the coracoid graft present (arrow) in the glenoid cavity. AP: Anteroposterior

The X-ray was suggestive of anterior dislocation of the right shoulder joint fixed by coracoid graft (Figure 2).

MRI Right Shoulder Joint

The MRI reports suggested that there was etiology of heterogeneous enhancement, and T2WI/PDF acts at hyper-intensities on the head of a humerus at the articular surface with erosion and adjacent soft tissue (rotator cuff tendons) enhancement.

**Figure 3:**
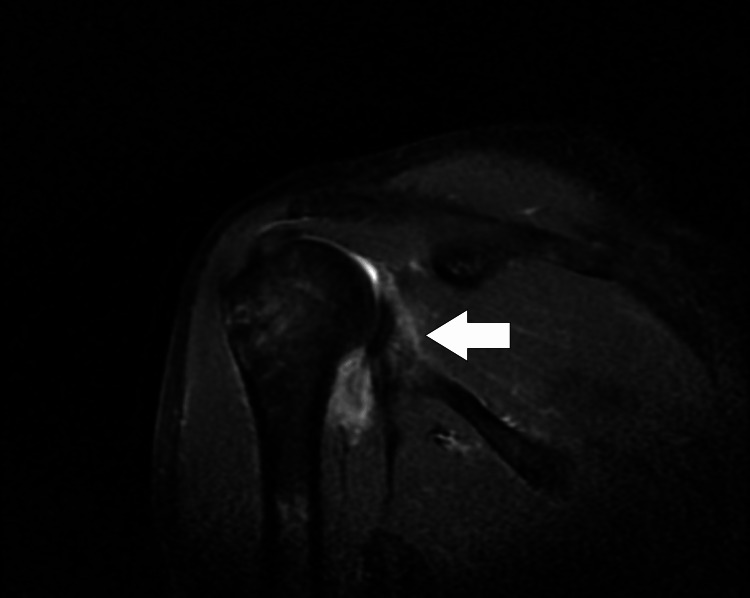
MRI of the right shoulder joint taken in the sagittal plane indicated by the arrow. MRI: Magnetic resonance imaging

**Figure 4:**
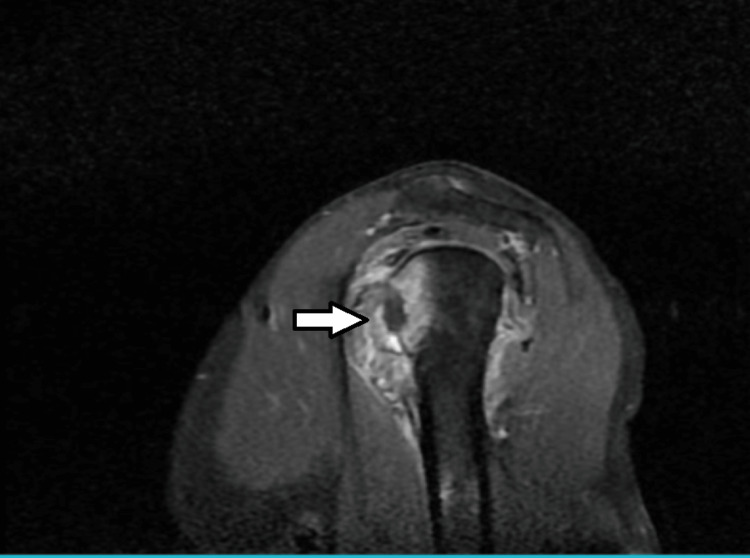
MRI of the right shoulder joint in the coronal plane indicated by the arrow. MRI: Magnetic resonance imaging

There is e/o fluid intensity collection in the glenohumeral joint space and sub-acromial bursa. There is an e/o peripherally enhancing collection in the subscapularis muscle. There is e/o heterogeneously enhancement, and T2WI/PDF acts at hyper-intensities in the deltoid, supraspinatus, infraspinatus, and subscapularis, muscles, and biceps tendon at its insertion. Therefore, the impression is seen in recurrent anterior dislocation of the right shoulder joint (Figures 3, 4).

Latarjet surgical intervention

Surgical Management 

The patient has undergone surgery for soft tissue reconstruction of the right shoulder joint with a Latarjet procedure. Regional block anesthesia was given, and the surgical approach was deltopectoral. Under the axillary block, the patient was taken to the OT table under aseptic condition in the beach-chair position. Painting, draping, and scrubbing were done on the right upper limb. The coracoid process was palpated and approximately 8 cm incision was made from the coracoid's tip inferiorly. The deltopectoral interval and cephalic vein were detected. The cephalic vein along with the deltoid is reflected laterally, and soft-tissue dissection was accomplished to expose the coracoid process. The coraco-acromial ligament was detached, and coracoid osteotomy was done with medial protection. Subscapularis and capsule were cut in a t-shaped fashion, and the Fukuda retractor was introduced in the joint space. Glenoid margins are exposed, and the coracoid process was fixed with two Herbert screws (3.5 mm x 30 mm^ -1 ^and 2.7 mm x 30 mm^ -1^ with suture anchor 5 mm^ -1^) over the anteroinferior border of the glenoid. Capsule and subscapularis were sutured back, and a drain was placed. The closure was accomplished in layers. A sterile dressing was applied. The skin closure suture was Ethilon 3-0.

Physiotherapeutic management

After the shoulder had been repositioned to its normal location, the rehabilitation phase, lasting approximately a year, was started. Exercise was part of physiotherapy care to repair modifiable physical deficiencies known to contribute to pain and dysfunction. Therapeutic exercise is commonly used in treating and managing a wide range of shoulder disorders, and it is highly suggested to address mobility, posture, muscular activation, proprioception, and strength dysfunctions.

Exercise Protocol 

To relieve pain before exercising, the patient was given analgesics and/or cold packs. Most patients experience aches, soreness, or stretching when performing these exercises. However, when the patient experienced acute and prolonged discomfort (for more than 30 minutes), it is a sign that the exercise should be modified to lessen intensity or duration. We used short, regular intervals (e.g. 5-10minutes, four times/day) instead of a single long practice. Repetition frequency was gradually increased. The patient was advised to follow the therapist's recommendations for the number of sets. The patient was told to get into the habit of doing the exercises in day-to-day life (Table 2).

**Table 2:** Physiotherapy protocol.

Intervention Phase-I (2-4 weeks)	Rationale	Dosage
Flexion of the shoulders.	To reduces stiffness within pain-free limits.	10 repetitions for three weeks.
Shoulder abduction.	To relieve stiffness and pain.	10 repetitions for three weeks.
Lateral rotation of the shoulder.	To prevent adhesion formation and stiffness.	10 repetitions for three weeks.
Phase II (4-6 weeks)		
Static strengthening exercises.	Static strengthening may help reduce pain and improve physical function.	Practice these exercises 10 times, withhold 5-10 seconds. Perform three times a day.
Mobility exercises.	Mobility is essential as it prepares the body for the stress of exercise.	Repeat each one 10 times, four times/day.
Scapular setting exercises.	In shoulder cases that restore its mobility, strength, and function, the shoulder blade must be properly positioned and supported.	Repeat 5-10 times each and hold for five seconds.
Techniques of strengthening the shoulders with a Theraband.	These exercises are performed with a Theraband as it is made to strengthen the muscles of the shoulder.	Each activity should be done 20 times, thrice a day withhold 3-5 seconds at the end range, and afterward, gently return to starting position.
Phase- III (6-8 weeks)		
Theraband exercises.	Resistance bands have been shown to increase muscular strength, endurance, and function in the elderly.	Repeat the exercises 10 times, three times a day, with a five-second hold.
Scapular setting exercises.	Scapular workouts strengthen the muscles that support the shoulder, thereby improving shoulder mobility and stability.	The patient has to hold the position for 5-10 seconds and relax.
Weight-bearing exercises.	These exercises are prescribed to strengthen the muscles that surround the shoulder joint.	Repeat 10 times.

Outcome measures

**Figure 5:**
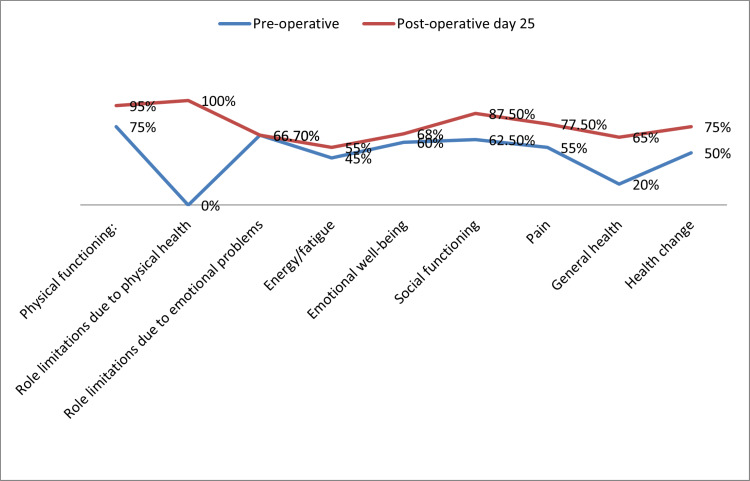
Health-related quality of life SF-36

The outcome measures are shown in Tables 3-5 and Figure 5.

**Table 3:** Manual muscle testing according to the Medical Research Council grading system.

Manual muscle testing According to the Medical Research Council grading system				
	Pre-operative on January 22, 2022		Post-operative on February 4, 2022	
Muscles	Right	Left	Right	Left
Shoulder				
Flexors	2/5	5/5	3/5	5/5
Extensors	2/5	5/5	3/5	5/5
Adductors	2/5	5/5	3/5	5/5
Abductors	2/5	5/5	3/5	5/5
Elbow				
Flexors	3/5	5/5	4/5	5/5
Extensors	3/5	5/5	4/5	5/5
Wrist				
Flexors	5/5	5/5	5/5	5/5
Extensors	5/5	5/5	5/5	5/5
Radial deviation	5/5	5/5	5/5	5/5
Ulnar deviation	5/5	5/5	5/5	5/5

**Table 4:** Patient range of motion in upper limb.

Range of Motion for Bilateral Upper Limb				
	Pre-operative on January 22, 2022		Post-operative on February 4, 2022	
Muscles:	Right	Left	Right	Left
Shoulder				
Flexion	85º	175 º	105 º	175 º
Extension	15º	45 º	25 º	45 º
Abduction	85º	175 º	105 º	175 º
Elbow				
Flexion	140º	155 º	150 º	155 º
Extension	140º- 0º	0 º- 155 º	0 º-150 º	0 º- 155 º
Wrist				
Flexion	85 º	90 º	90 º	90 º
Extension	65 º	70 º	70 º	70 º

**Table 5:** Oxford instability shoulder score.

Oxford instability shoulder score	
Pre-operative: 06/48 (poor)	Post-operative: 41/48 (excellent)

## Discussion

Research shows that rehabilitation of recurrent rotator cuff injuries in older people with catastrophic anterior shoulder dislocations that do not show injury to the anterior capsule labral structure can offer enough stability to permit normal life. However, if a shoulder dislocation occurs again after rotator-cuff restoration, the reconstructed rotator cuff may deteriorate or rupture again, making restoration more difficult [6]. As the anterior joint capsule is maintained, imbrications are not possible. To avoid recurrence, it is necessary to do an additional anterior structural repair that can replace the rotator cuff and capsule [7]. A study concluded that the Latarjet approach for addressing instability was described by Lafosse et al. in 1954 [8]. The long-term outcomes of this operation have been positive. According to a study, an all-arthroscopic approach for the Latarjet treatment was reported for the first time in 2007 [9]. In the United States, surgeons most frequently recommend the Latarjet treatment in situations of substantial glenoid bone loss, postoperative instability, and patients participating in high-risk sports. Another analysis concluded that after performing the Latarjet surgery on a patient with a shoulder dislocation [10]. A second study outlined the real clinical result of such a policy. In a randomized, prospective trial, 40 patients were placed into two groups: internal rotation and exterior rotation. The internal rotation group had a 30% dislocation recurrence rate compared with 0% in the external rotation group [11]. According to research, rehabilitation following the protocol entails a continuous progression; it takes four to six months to restore joint mobility as well as endurance with conserving bone reinforcement [12]. A study analyzing these statistics indicated that the Latarjet operation is a success, as shown by the great contentment of patients and minimal risk of repeated instability [13].

## Conclusions

The recurrent shoulder dislocation affects the muscle strength and has chances of ligament as well as tendon wear and tear. The above case demonstrates that a multidisciplinary team including physicians, orthopedic surgeons, and musculoskeletal physiotherapy treatment is profoundly found to be of utmost benefit for aiding symptoms like pain and improving functional abilities and the overall health of patients with recurrent shoulder dislocation. This case study demonstrated that effectiveness of musculoskeletal physiotherapy and the protocol planned was beneficial for the patient to perform his daily activities and work as soon as possible.
